# Supplementary figures and images for: A Comparative Analysis of a Self-Reported Adverse Events Analysis after Receiving One of the Available SARS-CoV-2 Vaccine Schemes in Ecuador
Source: Vaccines (Basel). 2022 Jun 30;10(7):1047. doi: 10.3390/vaccines10071047 (PMC9323750; doi:10.3390/vaccines10071047)

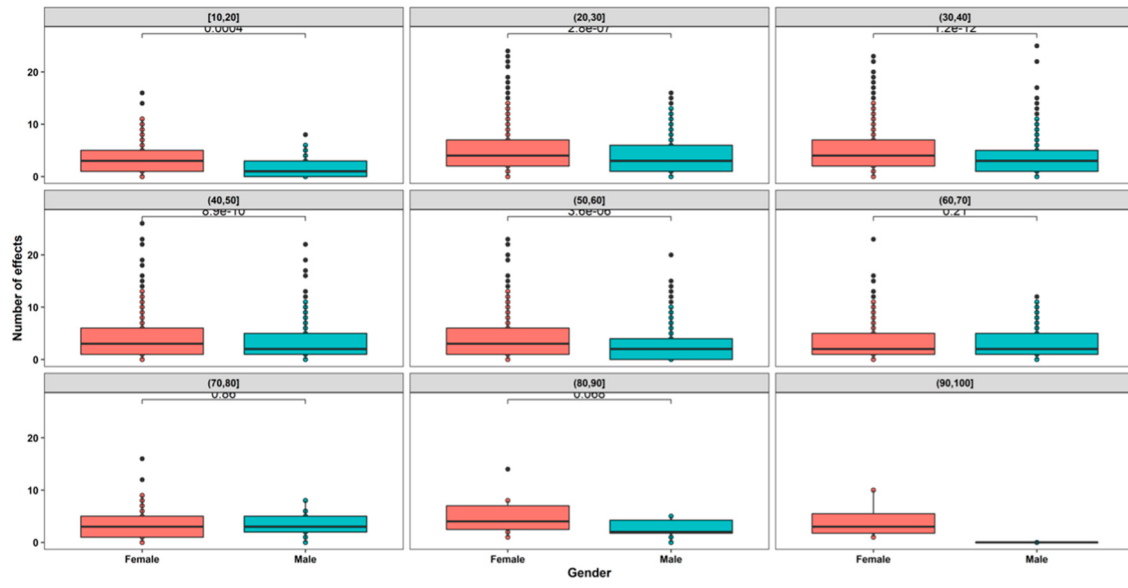

Supplement: Supplementary file 1 [file vaccines-10-01047-s001.zip › vaccines-1748086-supplementary.pdf]
